# The role of systemic chemotherapy and multidisciplinary management in improving the overall survival of patients with metastatic squamous cell carcinoma of the anal canal

**DOI:** 10.18632/oncotarget.2563

**Published:** 2014-11-14

**Authors:** Cathy Eng, George J. Chang, Y. Nancy You, Prajnan Das, Miguel Rodriguez-Bigas, Yan Xing, Jean-Nicolas Vauthey, Jane E. Rogers, Aki Ohinata, Priyanka Pathak, Salil Sethi, Jonathan K. Phillips, Christopher H. Crane, Robert A. Wolff

**Affiliations:** ^1^ Department of Gastrointestinal Medical Oncology, The University of Texas M.D. Anderson Cancer Center, Houston, TX 77030; ^2^ Department of Surgical Oncology, The University of Texas M.D. Anderson Cancer Center, Houston, TX 77030; ^3^ Department of Radiation Oncology, The University of Texas M.D. Anderson Cancer Center, Houston, TX 77030; ^4^ Division of Pharmacy, The University of Texas M.D. Anderson Cancer Center, Houston, TX 77030

## Abstract

Metastatic squamous cell carcinoma (SCCA) of the anal canal is a rare malignancy for which no standard treatment algorithm exists. To determine the best approach, all patients diagnosed with metastatic SCCA of the anal canal treated at a single institution were evaluated for choice of chemotherapy and treatment outcome. A retrospective study from January 2000 to May 2012 was conducted. Electronic medical records were reviewed for diagnosis of metastatic SCCA of the anal canal. All patients were treatment naïve for metastatic disease and completed all radiographic imaging at our institution. The purpose of this study was to evaluate outcomes among patients who received systemic chemotherapy and if appropriate were referred for multidisciplinary intervention (e.g., surgery, radiofrequency ablation, etc.). Seventy-seven patients fulfilled eligibility criteria. Forty-two patients (55%) received 5-fluorouracil (5-FU) + cisplatin (PF); 24 patients (31%) received carboplatin + paclitaxel (CP); 11 patients (14%) received an alternative regimen. After a median follow-up of 42 months, the median progression-free survival (PFS) for all patients was 7 months; the median overall survival (OS) was 22 months. Thirty-three patients (43%) underwent multidisciplinary management for metastatic disease resulting in a median PFS of 16 months (95% CI: 9·2 −22·8) and median OS of 53 months (95% CI: 28·3 – 77·6). Systemic chemotherapy provides durable survival for patients with surgically unresectable metastatic SCCA of the anal canal. Multidisciplinary management for select patients with metastatic disease effectively improves survival and should be considered whenever possible.

## NOVELTY AND IMPACT STATEMENT

Currently very little peer-reviewed literature exists regarding treatment of patients with metastatic squamous cell carcinoma of the anal canal. The purpose of this study, which is presently the largest of its kind, was to evaluate treatment outcomes among patients receiving multidisciplinary therapy and to evaluate the benefit of systemic chemotherapy as a treatment option which is being validated in the InterAACT trial. Our findings indicate that multidisciplinary management may provide an improvement in overall survival.

## INTRODUCTION

Carcinoma of the anal canal is a rare malignancy, representing only 2% of all digestive system cancers in 2014. Yet, the incidence of anal cancer continues to rise in the United States by 2% per year. Often considered a malignancy only associated with chronically immunosuppressed conditions, such as human immunodeficiency virus (HIV/AIDS) infection or transplant immunosuppression, it is commonly under recognized in non-immunosuppressed individuals. An estimated 7,210 new cases of anal carcinoma will be diagnosed in the United States, resulting in 950 deaths in 2014 [1]. Greater than 90% of the cases of carcinoma of the anal canal will be of squamous cell carcinoma (SCCA) origin. Other rare types of anal cancers include melanoma, adenocarcinoma, and neuroendocrine tumors. For the purpose of our analysis, we will only be discussing the more common SCCA of the anal canal.

The majority of patients with SCCA of the anal canal present with locally advanced disease but can be treated effectively with concurrent chemoradiation therapy reserving abdominal perineal resection (APR) only for surgical salvage therapy [2, 3]. Current standard treatment approaches for these patients include a definitive approach of concurrent chemoradiation consisting of 5-fluorouracil (5-FU) + mitomycin C or 5-FU + cisplatin. The adoption of this therapeutic approach for locally advanced SCCA of the anal canal has led to a 5-year overall survival ranging from 61%–85% [4–6, 11–13]. Yet, for those patients with recurrent locally advanced disease not amenable to APR or with distant metastatic disease, a median overall survival (OS) of only 8–12 months has been reported based in small case studies [7–10].

Extrapelvic metastases are diagnosed in only 5% of patients at the time of initial diagnosis, with metastatic disease occurring in 10%–20% of patients following treatment for locally advanced disease. Locally recurrent pelvic disease or distant disease can contribute significantly to patient morbidity and mortality as a result of local tumor effects such as pain, sacral involvement, symptomatic bulky necrotic lymphadenopathy, and destructive anal canal involvement.

Due to the low incidence of anal carcinoma and the rarity of the development of metastatic disease, there have been no completed prospective clinical trials exclusively for metastatic anal carcinoma. On the basis of anecdotal case reports and small retrospective case series [8–10, 14–16, 18–21], the general management of this disease in the metastatic setting has classically consisted of systemic chemotherapy. Yet, no chemotherapy treatment paradigm has been clearly established. Little published data exists other than small case studies or case series (Table 1), with minimal information regarding optimal management due to the limited sample size. Furthermore, prior retrospective studies suggested that there was no benefit in overall survival for surgical intervention in patients with metastatic SCCA [22]. Given the rising global incidence of anal carcinoma, it is likely more patients may be diagnosed with advanced disease. Furthermore, despite the use of anti-retroviral therapy in HIV+/AIDS patients, the incidence of anal carcinoma continues to rise [23]. Chemotherapeutic agents commonly utilized are those provided in other more common SCCA's: 5-fluorouracil (5-FU) and cisplatin, carboplatin, paclitaxel, and carboplatin and paclitaxel [24–29].

**Table 1:** Prior Case Reports/Cohorts in Metastatic SCCA of the Anal Canal

Author	N	Agents	ORR	Med PFS (months)	Med OS (months)
Wilking et al.^18^	15	Vincristine, Bleomycin, and High-dose Methotrexate	25%	2M	NR
Ajani et al.^19^	3	5-FU + Cisplatin	NA	17M (2 of 3)	NA
Faivre et al.^20^	18	5-FU + Cisplatin	65%(CR = 15%)	4M	NA
Hainsworth et al.^15^	60 (4 with squamous cell carcinoma of the anal cancer)	Docetaxel, Cisplatin, and 5-FU(max = 4 cycles)	65%(CR = 25%)	26M	NR
Jhawer et al.^9^	20	Mitomycin C, Adriamycin, Cisplatin, and Bleomycin-CCNU	12–20 (60%)	8M	15M
Alcindor^8^	5	Paclitaxel (1^st^ and 2^nd^ line)	60%	Range: 3–8M	Range: 4–20M
Abbas et al.^10^	7	Paclitaxel (2^nd^ line)	57%	Range: 2–8M	Range: 5–14M
Kim et al.^21^	8	Docetaxel, Cisplatin, and 5-FU	50% CR	Range: 19–88M	1 YR OS: 62.5%

ORR = objective response rate

PFS = progression free survival

OS = overall survival

NR = not reported

NA = not applicable

CR = complete response

The purpose of this study was to evaluate treatment outcomes among patients receiving systemic chemotherapy followed by multidisciplinary management or palliative systemic chemotherapy for metastatic SCCA of the anal canal.

## RESULTS

Seventy-seven patients met eligibility criteria for the purpose of this study. Baseline patient characteristics are described in Table 2 with subgroup demographics. All patients were evaluated for survival. The median age was 56 years. Only 7 patients (9%) had a known history of chronic immunosuppression (HIV or hepatitis). Six patients (8%) had a prior history of other sexually transmitted disease; 4 of those patients (5%) had a known history of HPV. The majority were female patients (70%). Fifty-two patients (67%) had received prior chemoradiation therapy for curative intent for early stage disease. In these select patients, the median interval from diagnosis to the development of metastatic disease was 19 months.

**Table 2:** Patient Demographics

	Total N = 77 (%)
Mean Age at Diagnosis of Metastatic Disease	56 (Range: 37–82)
Gender	
Male	23 (30)
Female	54 (70)
Histologic Grade	
Well	1 (1)
Moderate	26 (34)
Poor	39 (51)
Unknown	11 (14)
Prior Definitive Chemoradiation	
No	25 (33)
Yes	52 (67)
Prior History of Viral Infection:	
None	60 (78)
HIV	3 (4)
HPV	4 (5)
Hepatitis B or C	4 (5)
Other STD	6 (8)

HIV = human immunodeficiency virus

HPV = human papilloma virus

STD = sexually transmitted disease

The majority of tumor histology was moderate (34%) or poorly differentiated (51%). Frequent sites of metastatic disease included distant lymph nodes (33%), pelvis (31%), liver (25%), lungs (15%) and bone (8%).

Forty-two patients (55%) received 5-FU + cisplatin (PF); and twenty-four patients (31%) received carboplatin + paclitaxel (CP) as first line treatment; other therapy was provided in 11 patients (14%), (Table 3). Sixty-six patients (86%) were evaluable for response for the two most common regimens of PF and CP.

**Table 3:** Chemotherapy Regimens

Regimen	N = 77 (%)	Dosing Schedule
5-FU + Cisplatin (PF)	42 (55)	5-FU 750 mg/m2/day CI days 1–5 +Cisplatin 75 mg/m2 IV day 1, q28 Days
Carboplatin + Paclitaxel (CP)	24 (31)	Carboplatin AUC of 5 IV day 1 +Paclitaxel 175 mg/m2 IV day 1, q21 Days
Other	11 (14)	N/A

**Figure 1:**
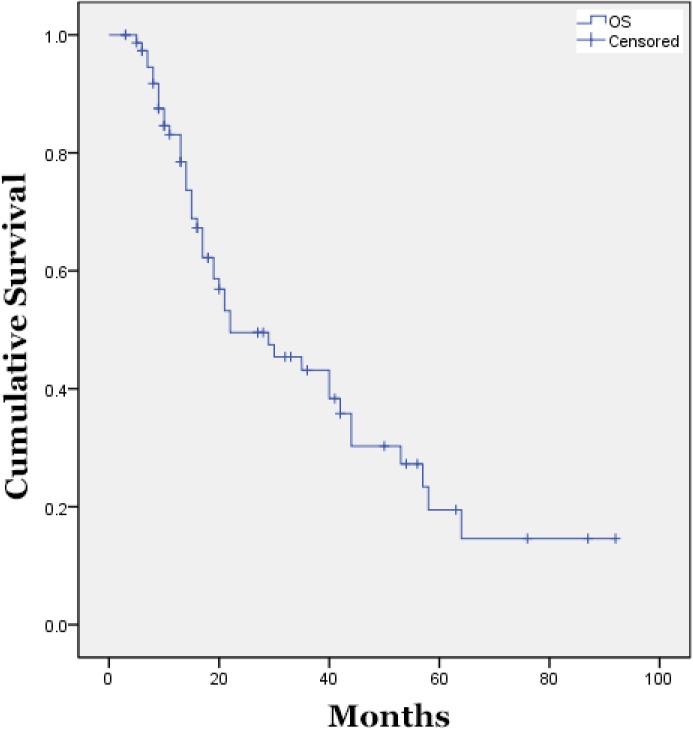
Median Overall Survival of All Treated Patients

After a median follow-up of 42 months, the median PFS for all patients was 7 months after receipt of a median of 4 cycles. The median OS was 22 months as indicated in Figure 1; the median number of subsequent lines of chemotherapy received was 1.

When stratified by most commonly provided chemotherapy regimens the observed median PFS was greater for PF vs. CP, 8 months (95% CI: 4·5 – 11·5) vs. 4 months (95% CI: 1·7 – 6·3), respectively, although this difference was not statistically significant. The response rate for the PF regimen also appeared more favorable (Table 4).

**Table 4:** Best Response – 5-FU + Cisplatin vs. Carboplatin + Paclitaxel

Regimen	N = 66 (%)
5-FU + Cisplatin (PF)	42 (63)
Stable Disease	12 (29)
Partial Response	24 (57)
Progressive Disease	6 (14)
Carboplatin + Paclitaxel (CP)	24 (37)
Stable Disease	5 (21)
Partial Response	8 (33)
Progressive Disease	11 (46)

Among the 33 patients who underwent multidisciplinary management for their metastatic disease following systemic chemotherapy, 19 (58%) underwent surgical resection of the metastatic site or RFA and 14 (42%) underwent chemoradiation, (Table 5). Of those patients undergoing surgical intervention, recurrent metastatic disease was specifically noted in the inguinal and retroperitoneal lymph nodes (N = 5, 22%), pelvis (N = 5, 22%), lung (N = 2, 8%), and liver (N = 11, 48%). Surgical intervention was provided as indicated in Table 5. If concurrent chemoradiation therapy was provided, PF was commonly used as a radiation sensitizer in 50% of patients. Subsequently, 4 of the 33 patients (12%) developed recurrent disease and underwent repeat surgical intervention [liver resection (N = 1); RFA of the liver (N = 1), and lung resection (N = 2)].

**Table 5:** Types of Multidisciplinary Treatment Provided to Patients

	N = 33 (43%)	
Gender	Male = 8 (24%)	Female = 25 (76%)
Treatments	Surgery	19 (58%)
	Chemoradiation	14 (42%)
Chemotherapy Regimen Provided During Radiation Therapy	5-FU + Cisplatin	7 (50%)
	5-FU + Mitomycin C	1 (7%)
	Carboplatin + Paclitaxel	2 (14%)
	5-FU or Capecitabine	2 (14%)
	None	1 (7%)
Surgical Treatments Provided	Liver Resection	9 (39%)
	Lung Resection	2 (9%)
	Lymph Node Dissection	5 (22%)
	Radical Pelvic Resection	4 (17%)
	Radiofrequency Ablation	3 (13%)
	Liver Resection	9 (39%)

**Figure 2:**
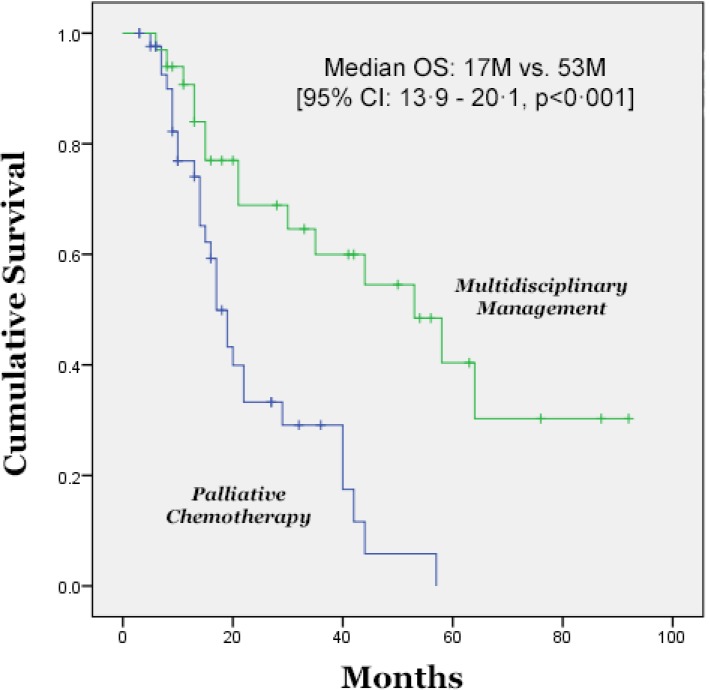
Median Overall Survival of Patients Treated with Multidisciplinary Intervention versus Palliative Systemic Chemotherapy

Of those patients who proceeded to receive multidisciplinary management for intent of cure, the median PFS was significantly longer at 16 months (95% CI: 9·2 – 22·8) compared to those patients receiving palliative systemic chemotherapy, with a PFS of only 5 months (95% CI: 3·5 – 6·5), p < 0·001. The difference in OS was also longer with a median OS of 53 months (95% CI: 28·3 – 77·6) compared to those undergoing palliative systemic chemotherapy, whose median OS was 17 months (95% CI: 13·9 – 20·1), p < 0·001 (Figure 2). When evaluating age, gender, race (white vs. non-white), only younger age was significant by univariate (*p* = 0·02) and multivariate analysis (*p* = 0·04) for increased overall survival.

## DISCUSSION

SCCA of the anal canal is viewed as an uncommon malignancy. Yet, the incidence continues to rise by 2% per year in the US and will impact greater than 27,000 individuals worldwide [30]. Little published data exists other than small case studies or case series, with minimal information for clinical derivation regarding optimal chemotherapy regimens or outcome due to the limited sample size. Here we present our experience in chemotherapy naïve patients who were deemed to be not amenable to salvage APR. The median age of our patient population was comparable to the general population with a median age of 56 years old. We determined the median interval for the development of metastatic disease following treatment for locally advanced disease to be less than 2 years indicating the importance of close surveillance during this period. We were able to establish that the use of 5-FU + cisplatin or carboplatin + paclitaxel therapy provides a reasonable benefit in PFS of 7 months and an OS of 22 months. Furthermore, our findings demonstrate the potential benefit of multidisciplinary management in select patients with a further improvement of PFS to 16 months and median OS of 53 months.

To our knowledge, this is the largest study to describe treatment outcomes in patients diagnosed with metastatic SCCA of the anal canal. Prior to our analysis the choice of chemotherapy has been 5-FU + cisplatin despite the fact that there is “limited data” (www.nccn.org). Furthermore, the NCCN guidelines suggest there is “no evidence to support surgical intervention” in metastatic anal cancer patients. Any consideration of additional types of chemotherapy for these patients has largely been informally extrapolated from the treatment of more common metastatic squamous cell carcinomas such as head and neck, lung, and cervical carcinoma [24–29]. Our data as presented here provides information regarding the role of 5-FU + cisplatin as well as carboplatin + paclitaxel. Here we also demonstrate the possible benefits of the utilization of a multidisciplinary approach when appropriate.

Our analysis is subject to several limitations. The data is retrospective and has been collected at a single institution which may result in an inherent treatment bias. 5-FU + cisplatin therapy was more commonly utilized overall and does not allow a direct comparison to carboplatin + paclitaxel. Carboplatin + paclitaxel was often chosen for palliation in unresectable patients likely as a result of the well-known potential treatment related toxicities associated with prolonged 5-FU + cisplatin therapy (e.g., nausea, vomiting, acute renal insufficiency, etc.). Due to the size of the study and retrospective nature of our analysis we were unable to differentiate a true difference between the two chemotherapy regimens for patients with unresectable disease. Chemotherapy dose intensity and dose delays also may not have been fully captured unless the treatment was provided directly at MDACC. The majority of patients were HIV- so the findings may not be generalizable to HIV+ patients.

We believe there are strengths to our analysis. A single institution analysis results in uniformity in care, permitting a more informative median PFS and OS evaluation than previously demonstrated in small case series. Furthermore, systemic chemotherapy supplemented by a multi-modality approach to optimize patient management provided patients an impressive median OS of 53 months which far exceeds previously reported median overall survival rates of 8–12 months in smaller case studies.

Though we are in the era of biologic agents or targeted therapies (i.e., bevacizumab, cetuximab, and panitumumab) the use of biologics was rarely provided in conjunction with chemotherapy (N = 2) due to patient provider concerns for reimbursement and due to the lack of prospective data for this patient population. However, recent phase III data indicate that cetuximab has a role in unresectable head and neck carcinomas in conjunction with radiation therapy and also for locally advanced, surgically unresectable, non-small cell lung carcinoma for response and overall survival [17, 31, 32]. The role of cetuximab as a radiation sensitizer has been investigated in combination with chemotherapy with curative intent for locally advanced anal cancer in HIV+ and HIV- patients [33, 34]. Both studies have completed accrual; final results are pending.

It has been previously reported that detectable human papilloma virus (HPV) has a favorable prognosis in head and neck cancer for disease-free and overall survival [35, 36]. A surrogate marker for HPV is p16, a cell cycle regulator [37]. The E7 oncoprotein of HPV may be inactivated by the retinoblastoma (Rb) protein resulting in p16 overexpression which is a positive prognostic factor for disease-free survival and overall survival in oropharyngeal cancer [38, 39]. We have recently evaluated the presence of the HPV positivity and its impact on prognosis in metastatic SCCA of the anal canal [40]. Our results suggest that HPV+ tumors have improved OS relative to HPV− tumors. However, the majority of patients (95%) were noted to have HPV+ tumors limiting our ability to adequately evaluate the HPV− tumor specimens. Post ad-hoc data from the phase III randomized SPECTRUM trial of PF +/− panitumumab indicate that HPV positivity was associated with a survival benefit when combined with fully human monoclonal antibody, panitumumab, thereby indicating further analysis is warranted regarding HPV positivity and role of anti-EGFR therapy [41, 42].

It is presumed the incidence of anal carcinoma will continue to gradually increase over the next several years. Given the rising incidence of anal carcinoma, it is likely more patients will be diagnosed with advanced disease. Globally, HPV remains the most common sexually transmitted disease [43]. Despite the advent of anti-retroviral therapy in HIV+ patients and the increased life expectancy of the HIV patient population, the incidence of SCCA of the anal canal has not decreased [44, 45]. Recent approval for the quadrivalent HPV vaccine was granted in the US for adolescent/teenage girls as well as sexually active woman up to 26 years of age for the prevention of cervical cancer [46, 47], yet its use has not been widely adopted in the US, and its role in the treatment of carcinoma of the anal canal is investigational. Given the current available data in the non-HIV and HIV+ patient population, it is clear further exploration of chemotherapy with or without the addition of biologic agents should be formally pursued for the metastatic anal carcinoma patient population.

With the support of the National Cancer Institute, the International Rare Cancer Initiative (IRCI) recognizes the global impact of anal carcinoma and the need to identify the most appropriate treatment regimen for metastatic SCCA of the anal canal. InterAACT (NCT02051868) is the first prospective randomized phase II study ever to be conducted in metastatic SCCA of the anal canal patients and will compare 5-FU + cisplatin versus carboplatin + paclitaxel. The trial is now open to enrollment [48]; HIV+ patients will be considered eligible. Tissue correlatives will be collected in InterAACT. With the successful completion of this study, it is the hope that biologic agents will be studied in the phase III setting.

## CONCLUSIONS

To date, there is no established prospectively evaluated chemotherapy algorithm for metastatic squamous cell carcinoma of the anal canal. Based on our findings, 5-FU + cisplatin or carboplatin + paclitaxel regimens have activity in the treatment of metastatic SCCA of the anal canal warranting further analysis. More importantly, multidisciplinary management (e.g., surgical resection, RFA, stereotactic radiation therapy, etc.) should be considered for select patients whenever appropriate to improve their overall outcomes.

## MATERIALS AND METHODS

Patients with metastatic SCCA of the anal canal treated at MD Anderson Cancer Center (MDACC) between January 1, 2000 and May 1, 2012 were identified. All patients were required to be treatment naïve to systemic chemotherapy for metastatic disease but may have received prior definitive chemoradiation therapy for their locally advanced primary tumor. All patients were reviewed by the MDACC colorectal surgical oncology team and were determined to not be amenable to surgical salvage with an abdominal perineal resection (APR). All patients were recommended to proceed with systemic chemotherapy. Medical records were reviewed for patient demographics, tumor characteristics, and clinical outcomes including: progression-free survival (PFS), overall survival (OS), response rate (RR), prior history of sexually transmitted diseases (STDs) or immunosuppressed state (if documented), histology, chemotherapy regimen, subsequent lines of therapy, and treatment intervention. All patients were provided treatment recommendations by an MD Anderson physician but were allowed to receive their systemic chemotherapy at an outside institution for added convenience. All patients were required to have all radiographic imaging including a CT scan of the chest, and CT or MRI of the abdomen, and pelvis at our facility as well as follow up. If systemic treatment was provided at an outside institution, corresponding physician notes were utilized for verification of toxicity, and response to the recommended treatment regimen. If the patient had demonstrated partial response/stable disease from their systemic chemotherapy, the patient was referred for multidisciplinary treatment recommendations to either surgical oncology or radiation oncology for consultation.

For accuracy, we limited the sample to include only those patients who were initially evaluated with treatment recommendations provided at MDACC plus at least 1 follow-up visit with a physician or a mid-level provider with restaging diagnostic imaging completed at MDACC. Patients with prior or concurrent malignancies within 5 years of diagnosis, except squamous or basal cell skin carcinomas, were excluded.

Two treatment categories were created: 1) Palliative systemic chemotherapy was defined for all patients with diffuse metastatic disease not amenable to surgical or radiation oncology intervention, or 2) Multidisciplinary management inclusive of those patients that had received systemic chemotherapy but who were determined by the multidisciplinary team to potentially benefit from treatment intervention (e.g., surgery, stereotactic radiation therapy, radiofrequency ablation, etc.).

OS was defined as date of diagnosis of metastatic disease until date of death or last follow-up. PFS was defined as date of initiation of therapy until date of documented progression. Follow-up was performed by review of clinic notes and contact with corresponding outside oncologist. Death and overall survival were also determined by use of the social security death index (SSDI) search, which was corroborated with institutional medical records and the tumor registry of MDACC. The distribution of each continuous variable was summarized by its mean, standard deviation and range. The distribution of each categorical variable was summarized in terms of its frequencies and percentages. Log rank tests were used to compare each time-to-event variable (OS and PFS) between groups using the Kaplan-Meier method. This analysis was approved by the institutional review board at MDACC and a waiver of consent was granted.
